# Defective Neurogenesis in Lowe Syndrome is Caused by Mitochondria Loss and Cilia-related Sonic Hedgehog Defects

**DOI:** 10.1101/2024.11.01.621496

**Published:** 2024-11-01

**Authors:** Chien-Hui Lo, Siyu Chen, Jingyu Zhao, Zhiquan Liu, Biao Wang, Qing Wang, Tia J. Kowal, Yang Sun

**Affiliations:** 1 Department of Ophthalmology, Stanford University School of Medicine, Palo Alto, CA, USA; 2 Palo Alto Veterans Administration, Palo Alto, CA, USA; 3 Maternal Children Health Research Institute at Stanford, Stanford University School of Medicine, Palo Alto, CA, USA; 4 BioX, Stanford University School of Medicine, Palo Alto, CA, USA

**Keywords:** Neuronal differentiation, Cilia formation, Mitochondria, Lowe Syndrome, OCRL

## Abstract

Human brain development is a complex process that requires intricate coordination of multiple cellular and developmental events. Dysfunction of lipid metabolism can lead to neurodevelopmental disorders. Lowe syndrome (LS) is a recessive X-linked disorder associated with proximal tubular renal disease, congenital cataracts and glaucoma, and central nervous system developmental delays. Mutations in OCRL, which encodes an inositol polyphosphate 5-phosphatase, lead to the development of LS. The cellular mechanism responsible for neuronal dysfunction in LS is unknown. Here we show depletion of mitochondrial DNA and decrease in mitochondrial activities result in neuronal differentiation defects. Increased astrocytes, which are secondary responders to neurodegeneration, are observed in neuronal (iN) cells differentiated from Lowe patient-derived iPSCs and an LS mouse model. Inactivation of cilia-related sonic hedgehog signaling, which organizes the pattern of cellular neuronal differentiation, is observed in an OCRL knockout, iN cells differentiated from Lowe patient-derived iPSCs, and an LS mouse model. Taken together, our findings indicate that mitochondrial dysfunction and impairment of the ciliary sonic hedgehog signaling pathway represent a novel pathogenic mechanism underlying the disrupted neuronal differentiation observed in LS.

## Introduction

Lowe syndrome (LS) (OMIM #309000) is a rare X-linked disorder characterized by bilateral congenital cataracts and glaucomatous optic nerve degeneration, renal tubular dysfunction, and intellectual disability (Murdock and Chou, 2024; Nusbaum et al., 2015; Schaub et al., 2017; Shah et al., 2024). Mutations in the oculocerebrorenal syndrome of Lowe (OCRL) gene are responsible for Lowe syndrome. This gene encodes an inositol polyphosphate 5-phosphatase that preferentially cleaves phosphatidylinositol 4,5-bisphosphate (PI_(4,5)_P_2_) to generate PI_4_P (Lewis et al., 1993; Luscher et al., 2019; Prosseda et al., 2017; Sakakibara et al., 2022; Sharma et al., 2015). Neurologically, patients with LS often exhibit developmental delays, intellectual disability, absent deep tendon reflexes, and hypotonia (Bökenkamp and Ludwig, 2016; Lewis et al., 1993). Seizures and behavioral issues such as hyperactivity and aggression are also common and difficult to treat. MRI findings frequently show structural brain abnormalities, including dilated periventricular spaces and small cystic lesions, in later stages of the disease. However, the differential diagnoses in LS are broad and include diverse metabolic and neuronal disorders, especially when neurologic impairment is prominent. Importantly, recent case reports have suggested the importance of mitochondrial dysfunction in the pathogenesis of LS. For example, a 5-year-old boy with LS caused by OCRL mutation was initially diagnosed as a mitochondriopathy with electron microscopic evidence of mitochondrial changes (Dumic et al., 2020). Another patient suspected to have chronic progressive external ophthalmoplegia (CPEO) due to mitochondrial disease showed a missense mutation in OCRL (Ali et al., 2024; Craigen et al., 2013; Eliyan et al., 2023). Despite these isolated reports of mitochondrial defects in OCRL mutated patients, no clear mechanism explains the neurological anomalies in LS.

Increasing evidence shows that neuron stem cell (NSC) differentiation toward either the neuronal or astroglial lineage is determined by reactive oxygen species (ROS) levels (Adusumilli et al., 2021; Shahin et al., 2023). Furthermore, mtDNA integrity and mitochondrial complex I activity are involved in the NSC differentiation, suggesting that mitochondrial damage is one of the first signals for elevated astrogliosis and decreased neurogenesis during pathological development of the central nervous system (CNS) and after neuronal injury (Ignatenko et al., 2018; Wang et al., 2011).

Astrocytes play an essential role in maintaining ionic balance, blood-brain barrier integrity, synapse function, and metabolic homeostasis in the CNS (Cabezas et al., 2014; McNeill et al., 2021; Oksanen et al., 2019; Pociūtė et al., 2024). In response to CNS injury, disease, or infection, they undergo a diverse array of morphological, molecular, and functional changes that are referred to as reactive astrogliosis (Escartin et al., 2021; Matusova et al., 2023; Zamanian et al., 2012). Despite their crucial role in CNS metabolism, however, it remains unclear how they are affected by metabolic stress. Previously we discovered increased astrogliosis in the neuronal retinal cells in the eye in LS.

Here, we hypothesize that mitochondrial dysfunction may cause defective neurogenesis in LS with differentially regulated neuronal-astrocyte development. To test this hypothesis, we used a rapid single-step procedure to convert induced pluripotent stem cells (iPSCs), derived from a LS patient, into neurons (iN) and determined whether the failure of mitochondria to meet the energy required for normal CNS development accounts for impaired neuronal differentiation in this illness.

## Results

### Distinct differentiation of neuronal stem cells and neuronal progenitor cells (NSPCs) in OCRL knockout and LS iPSCs

**Figure 1.**
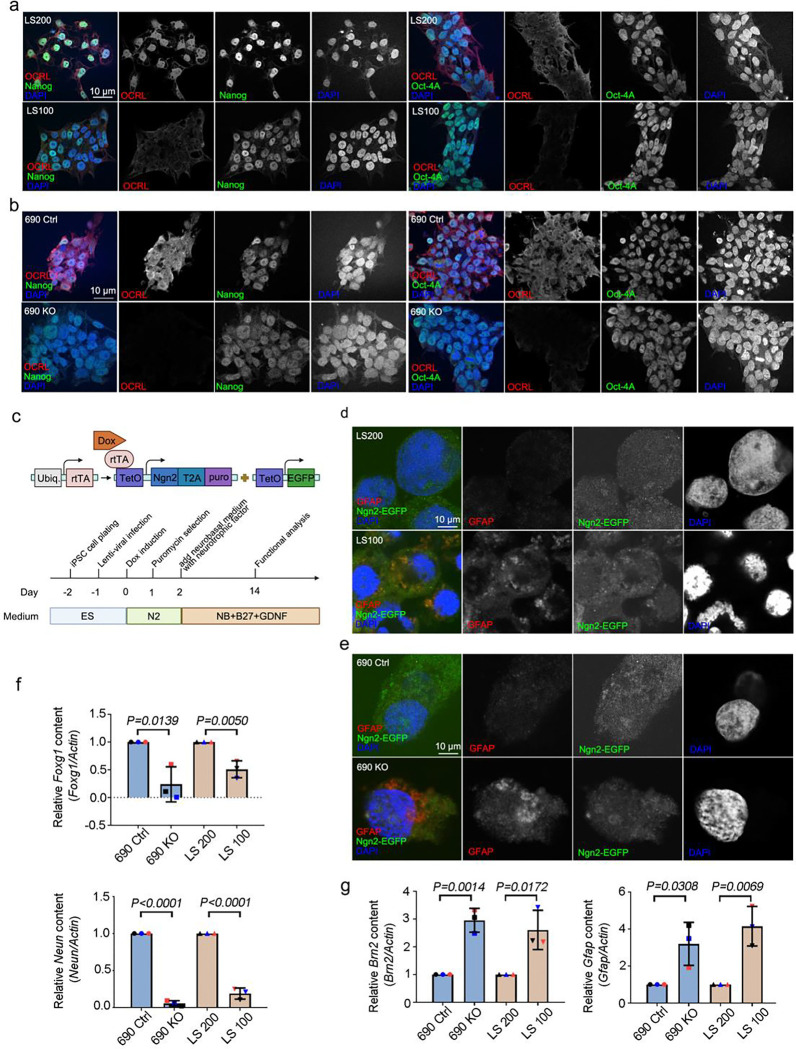
Increased astrocyte production during neuronal differentiation in OCRL-deficient and Lowe syndrome iPSCs. (a and b) Lowe syndrome iPSCs and *OCRL* knockout iPSCs stained with *OCRL* (red) and *Nanong; OCT4* (green) antibodies. DNA stained with DAPI (blue). Scale bars are as indicated. (c) Design of lentiviral vectors to induce Ngn2-mediated conversion of iPSCs to neuronal (iN) cells. (d and e) Lowe syndrome iPSCs and *OCRL* knockout iPSCs stained with GFAP (red) antibodies and expressed Ngn2-EGAP. DNA stained with DAPI (blue). Scale bars are as indicated. (f) Quantitative real-time PCR (RT-PCR) validation of *FOXG1* and *NEUN* in iN cells. RT-PCR was repeated three times with different batches. Gene expression values are normalized to GAPDH (g) Quantitative real-time PCR (RT-PCR) validation of *BRN2* and *GFAP* in iN cells. RT-PCR was repeated three times with different batches. Gene expression values are normalized to *GAPDH*. The bars in each graph represent mean ± SD. Statistical significance was determined using Student’s t-test, with exact p-values reported.

LS patients with *OCRL* mutations develop a wide range of neurologic disorders (Ramadesikan et al., 2021). To determine whether *OCRL* mutations influence neuronal development, we developed an *in vitro* system of functional induced neuron (iN) cells differentiated from Lowe syndrome patient-derived iPSCs (Zhang et al., 2013). Patient-specific iPS cells came from a boy with LS; familial controls came from his normally developing brothers. LS 100 is a 17-year-old with Lowe syndrome who was delivered after a 36-week pregnancy and diagnosed by genetic sequencing of OCRL mutations. LS200 is his older brother who was 22 years old when he provided blood for iPS cell generation (Barnes et al., 2018). In addition to the patient- and sibling - originated iPSCs, we used iPSCs produced from two sources by gene editing, including an OCRL KO line (690 KO) generated using CRISPR-Cas9 gene editing from a previously described control (690 Ctrl) that was unrelated to the LS subjects (Barnes et al., 2018; Ran et al., 2013). We applied the pluripotency markers, Nanog and Oct-4a, to our iPSCs to validate their stemness and proliferative potential. Our *OCRL* knockout and *OCRL* mutation iPSC models showed highly efficient proliferation and stemness (Figure 1a and b). No *OCRL* protein expression was observed in *OCRL* knockout iPSCs; *OCRL* protein expression was markedly lower in LS-patient derived iPSCs (LS 100) than in WT-*OCRL* expressing iPSCs (LS 200) (Barnes et al., 2018). We used the rapid single-step method to convert iPSCs into iN cells (Figure. 1c) (Ran et al., 2013). To identify the cellular processes controlling the differentiation of NSPCs into neurons and astrocytes, we used immunostaining to verify the stages of neuron differentiation. We found more astrocytes among cells originating in *OCRL* knockout and LS-patient derived iPSCs than in those from wild-type and control iPSCs (Figure 1d and e). We also used qPCR to assess the expression of individual genes characteristic of neurons and astrocytes, *FOXG1* (neurons), *NEUN* (neurons), *BRN2* (astrocytes) *and GFAP* (astrocytes). Surprisingly, we discovered that levels of the astrocytic genes in *OCRL* knockout and mutations iPSCs were markedly higher than in control cells, whereas, in comparison, neuron-specific genes were highly expressed in wild-type and control iPSCs (Figure 1f and g). Therefore, we conclude that OCRL - deficient neuronal progenitor cells preferentially favor the astrocytic lineage rather than the neuronal lineage.

### Decreased functional mitochondrial activities during neurogenesis in OCRL knockout and LS-patient derived iN cells

**Figure 2.**
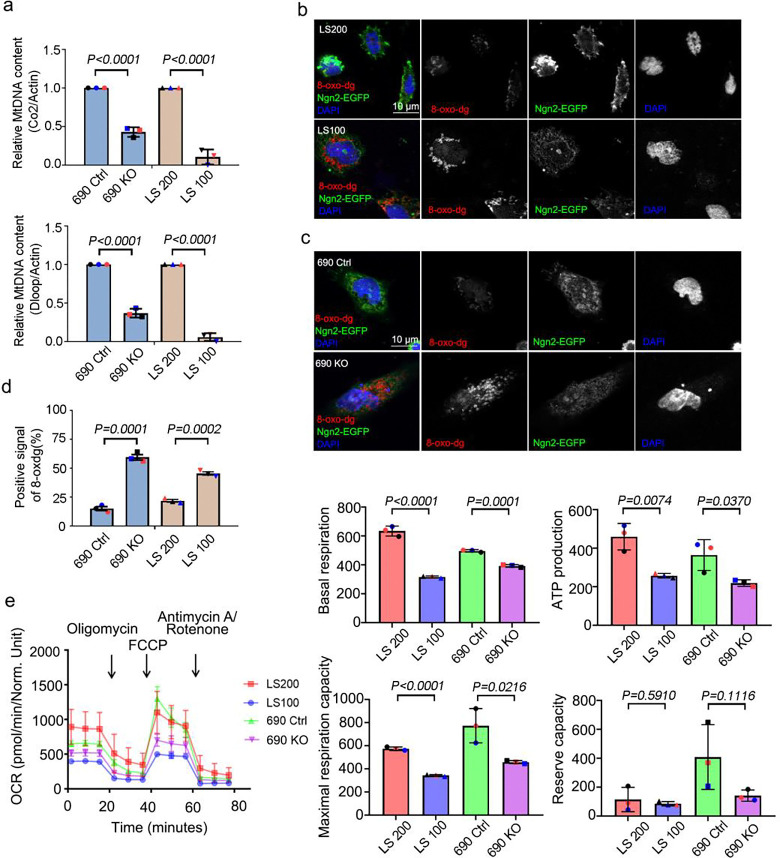
Mitochondria defects in iN cells derived from OCRL-deficient iPSCs. (a) Quantitative real-time PCR (RT-PCR) validation of mitochondrial DNA genes *COX2* and *DLOOP* in iN cells. RT-PCR was repeated three times with different batches. Gene expression values are normalized to *ACTIN*. (b and c) iN cells derived from Lowe syndrome iPSCs and *OCRL* knockout iPSCs stained with 8-oxo-dg (red) antibodies and expressed Ngn2-EGAP. DNA stained with DAPI (blue). Scale bars are as indicated. (d) Quantification of the percentage of iPSCs-derived iN cells positive for 8-oxo-dg signal. > 100 cells analyzed for each independent experiment. (e) Oxygen consumption rate of Lowe syndrome iPSCs-derived iN cells and OCRL knockout iPSCs-derived iN cells measured by Seahorse Analyzer. The bars in each graph represent mean ± SD. Statistical significance was determined using Student’s t-test, with exact p-values reported.

Mitochondrial dysfunction is a hallmark of neurodegenerative diseases, including progressive Parkinson’s disease (Bantle et al., 2020; Klemmensen et al., 2024), and is known to impact astrocytes (Ignatenko et al., 2023). Based on the clinical observation of mitochondrial defects in LS kidney samples, we hypothesized that OCRL deficiency may lead to mitochondrial defects in neurons. To test whether mitochondrial activity is defective during neurogenesis in LS, we examined mitochondrial activities in OCRL knockout and LS-patient-derived iN cells. Using qPCR, we measured mtDNA gene expression, *COX2* and *DLOOP* and found markedly decreased mtDNA gene expression in OCRL knockout and LS-patient-derived iN cells as compared to wild-type and controlled iN cells (Figure. 2a). Assessment of oxidative stress in these cells using immunostaining of 8-oxo-dg revealed positive oxidative stress signals in OCRL knockout and LS-patient-derived iN cells as compared to wild-type and controlled iN cells (Figure. 2b, c and d). Because these results suggested that mitochondrial oxidative phosphorylation (OXPHOS) is defective in LS, we examined the functional properties of mitochondria during neurogenesis, focusing on mitochondrial OXPHOS levels. In a parallel approach to assess mitochondrial function, we also measured the mitochondrial oxygen consumption rate (OCR) using seahorse metabolic profiling. We discovered that mitochondrial OCR was significantly lower in OCRL knockout and LS-patient-derived iN cells than in wild-type and non-affected sibling control iN cells (Figure 2e).

### Elevated astrocytic reaction during the differentiation of NSPCs in the Lowe syndrome (IOB) mouse model

**Figure 3.**
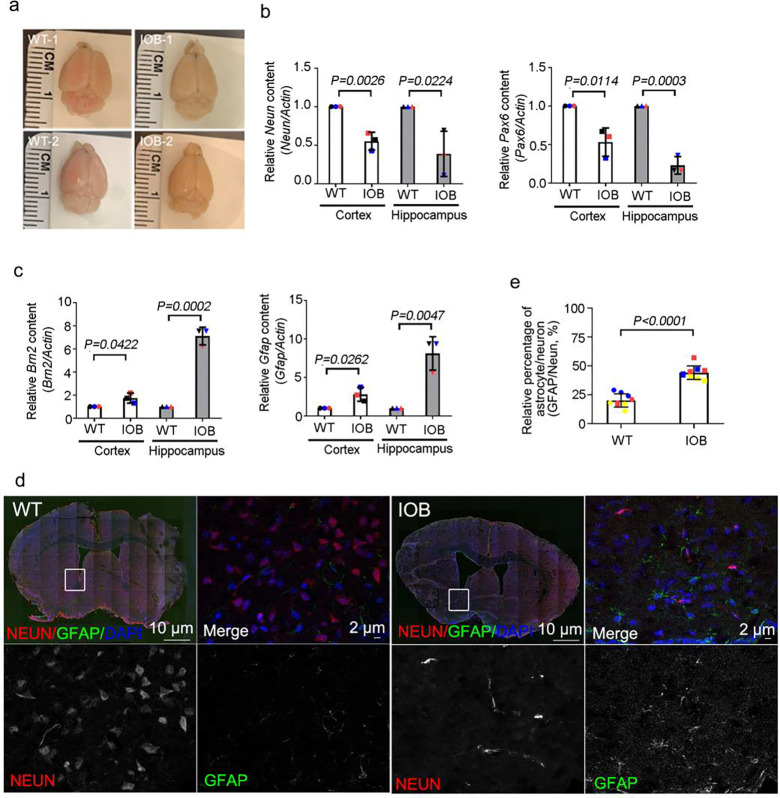
Elevated astrocyte population during neuronal differentiation in Lowe syndrome mouse model. (a) Images showing brain of wild-type and IOB mouse. (b) Quantitative real-time PCR (RT-PCR) validation of *Neun* and *Pax6* in brain sections. RT-PCR was repeated three times with different batches. Gene expression values are normalized to actin. (c) Quantitative real-time PCR (RT-PCR) validation of *Brn2* and *Gfap* in brain tissues. RT-PCR was repeated three times with different batches. Gene expression values are normalized to actin. (d) Wild-type and IOB mouse brain section stained with Neun (red) and GFAP (green) antibodies. DNA stained with DAPI (blue). Scale bars are as indicated. (e) Quantification of the ratio of Neun and GFAP signals in brain section(s) of wild-type and IOB mouse. > 100 cells analyzed for each independent experiment. The bars in each graph represent mean ± SD. Statistical significance was determined using Student’s t-test, with exact p-values reported.

Based on our iPSC models, we hypothesized that CNS development in the LS mouse model would exhibit higher levels of astrocytic progenitor cells than neuronal progenitor cells. We previously described the ocular phenotype of the humanized Lowe syndrome (IOB) mouse model. Here, we examined the differentiation of NSPCs into neurons and astrocytes in brain sections of the IOB mouse model (Supplementary figure 3) (Bothwell et al., 2011). Brain size was smaller in IOB mice than in WT mice (Figure 3a). We examined the gene expression of neurons (*Neun*, *Pax6*) and astrocytes (*Brn2*, *Gfap)* and discovered more gene expression of astrocytic genes in brain sections of IOB than WT mice at the same age cohort (Figure. 3b and c). We also examined the outcome of NSPC differentiation in brain sections of IOB and WT and found that the ratio of astrocytes was higher in IOB than in WT (Figure. 3d and e). Therefore, we concluded that, as in the iPSC cell models of LS, the IOB mouse model for LS also shows greater differentiation into astrocytes than WT controls.

### Decreased levels of mitochondrial function in CNS development in LS mouse model.

**Figure 4.**
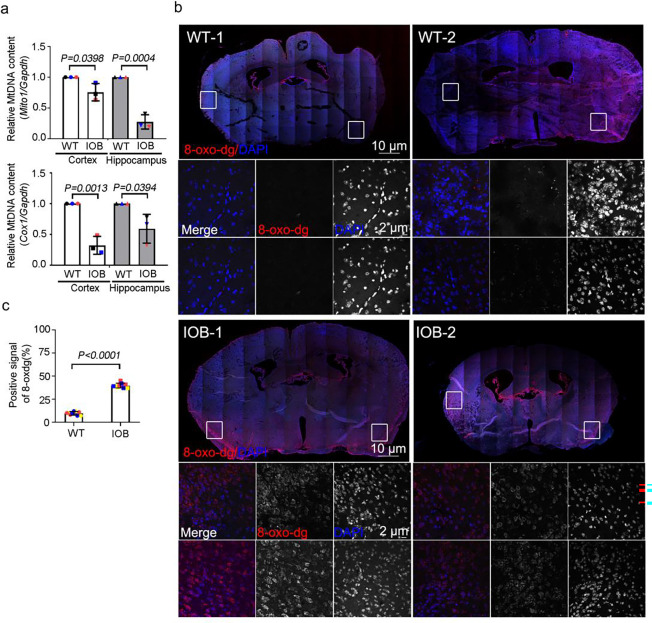
Mitochondrial defects in the brain of Lowe syndrome mouse model. (a) Quantitative real-time PCR (RT-PCR) validation of mitochondrial DNA genes *Cox2* and *Dloop* in brain sections of wild-type and IOB mouse. RT-PCR was repeated three times with different batches. Gene expression values are normalized to Actin. (b) Brain sections of wild-type and IOB mouse stained with 8-oxo-dg (red) antibody. DNA stained with DAPI (blue). Scale bars are as indicated. (c) Quantification of the positive percentage of 8-oxo-dg signals in brain sections of wild-type and IOB mouse > 100 cells analyzed for each independent experiment. The bars in each graph represent mean ± SD. Statistical significance was determined using Student’s t-test, with exact p-values reported.

We further assessed whether changes in mitochondrial activity are involved in the altered differentiation of stem cells and progenitor cells in the LS mouse model. Based on the metabolic studies on LS-patient derived cells, we hypothesized that OCRL deficient LS mouse model would similarly show altered mitochondrial functions. Examination with qPCR revealed less expression of the mtDNA genes *mito1 and cox1* in brain tissues of IOB than WT (Figure. 4a). We also assessed the oxidative stress levels in OCRL deficient and WT brain sections by measuring 8-oxo-dg, a validated measure for oxidative damage. Positive oxidative stress signals were significantly higher in the brain sections of 3-month-old IOB mice than of WT mice of the same age (Figure 4b and c). Therefore, we concluded that the brains of the OCRL-deficient LS mouse model, in which levels of astrocytes but not neurons are increased, also demonstrate an increased level of mitochondrial oxidative stress and decreased mtDNA levels.

### Defective ciliary homeostasis underlies neuronal dysfunction in Lowe syndrome

**Figure 5.**
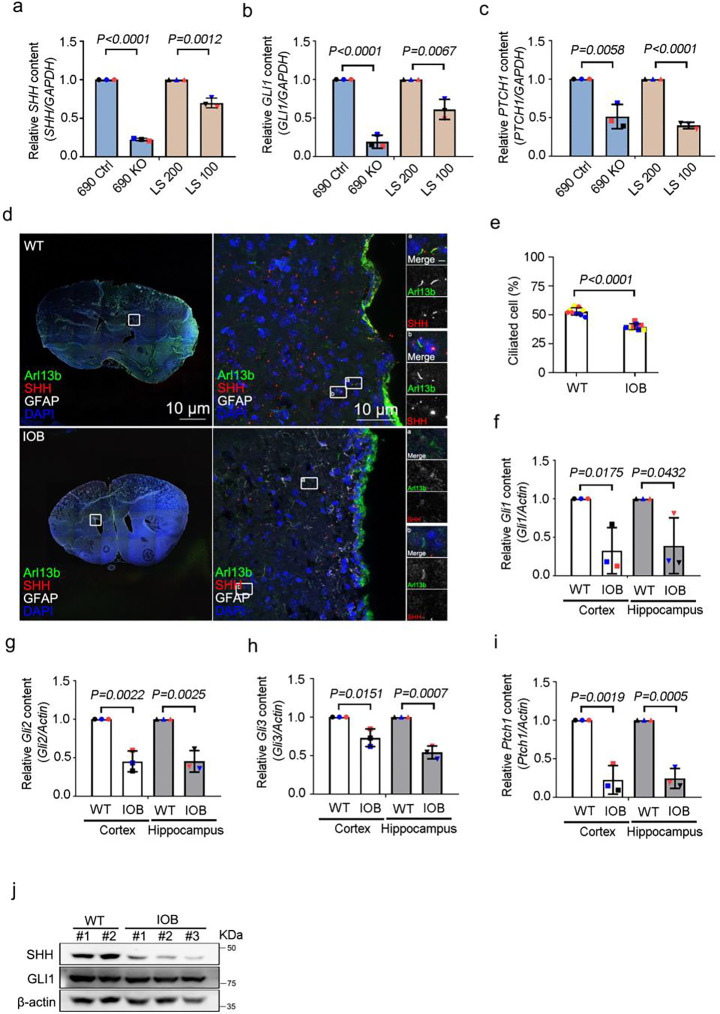
Involvement of cilia-mediated Shh signaling in neuronal differentiation of OCRL-deficient iN cells. (a, b and c) Quantitative real-time PCR (RT-PCR) validation of Shh signaling genes *SHH*, *GLI1* and *PTCH1* in iN cells. RT-PCR was repeated three times with different batches. Gene expression values are normalized to *GAPDH*. (d) Brain section of wild-type and IOB mouse stained with SHH (red) and Arl13b (green) antibodies. DNA stained with DAPI (blue). Scale bars as indicated. (e) Quantification of the percentage of positive ciliated cells. > 100 cells analyzed for each independent experiment. (f, g, h and i) Quantitative real-time PCR (RT-PCR) validation of Shh signaling genes *Gli1, Gli2, Gli3 and Ptch1* in brain section**s** of wild-type and IOB mouse. RT-PCR was repeated three times with different batches. Gene expression values are normalized to *actin*. (j) Western blot analysis using antibodies against SHH, GLI1 and β-actin in brain sections of wild-type and IOB mouse. The bars in each graph represent mean ± SD. Statistical significance was determined using Student’s t-test, with exact p-values reported.

Mitochondrial defects have been shown to disrupt ciliary homeostasis in astrocytes (Ignatenko et al., 2023). The primary cilium plays a crucial role in Hedgehog (Hh) signal transduction, which is essential for the proper development and function of the central nervous system and neural progenitor cells (Ho and Stearns, 2021; Nozawa et al., 2013). Here, we determined the sonic hedgehog (Shh) ciliary signal, which is involved in neuronal differentiation, in *OCRL* knockout iN cells and *OCRL* mutation iN cells. We used qPCR to assess genes involved in the Shh pathway, including *SHH, GLI1* and *PATCH1.* Shh signaling was markedly lower in *OCRL* knockout and LS-patient derived iN cells than in wild-type and unaffected LS sibling-derived iN cells (Figure. 5a, b and c). We also assessed cilia formation in brain sections of IOB and WT and found defective cilia formation in those of IOB (Figure 5d and e). We also examined mRNA levels with qPCR to determine whether the Shh pathway is affected during neurogenesis in LS. Measurements of mRNA for *Gli1, Gli2, Gli3* and *Ptch1* demonstrated that levels of Shh signaling pathway genes were reduced in the brains of IOB mice compared to those of WT mice (Figure. 5f–i). Protein levels of Shh and Gli1 were also significantly decreased in IOB brain compared to WT brain (Figure. 5j).

**Figure 6.**
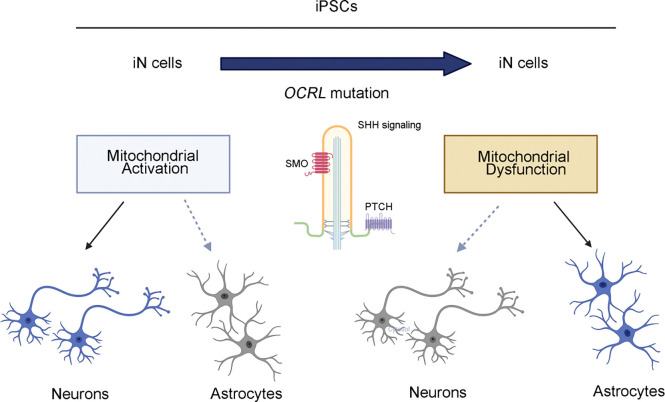
Mitochondrial-mediated neuronal differentiation defects in OCRL-deficient Cells. Neuronal cells induced (iN) from iPSCs derived from mutant OCRL and Lowe syndrome mouse models contain a high level of astrocytes. OCRL knockout, mutant iPSCs-derived iN cells, and Lowe syndrome mouse model also possess reduced cilia-related sonic hedgehog pathways (created by BioRender.com).

Taken together, the results of the present studies of iPSCs derived from an LS patient and the IOB mouse model of LS show that mitochondrial activities, mtDNA loss and ciliary homeostasis are involved in neuronal development in Lowe syndrome. We also show an increase of astrocytes but not of neurons in the IOB mouse model of Lowe syndrome brain and neuronal cultures that supports a novel role of OCRL as a critical switch for controlling neuronal-astrocyte differentiation: the loss of OCRL results in a decreased neuron: astrocyte ratio (Figure. 6). In addition, our findings present evidence of mitochondrial dysfunction in neuronal development in Lowe syndrome model systems and reveal a link between mitochondria and cilia signaling. This research offers a therapeutic perspective for patients with Lowe syndrome.

## Discussion

Lowe syndrome, an X-linked disorder caused by OCRL mutations, leads to neurodevelopmental delays and other issues. Here, we showed that a depletion of mitochondrial DNA and a decline in mitochondrial activity is associated the altered differentiation, leading to defects in the differentiation of neuronal cells. We found that induced neuronal (iN) cells derived from mutant-OCRL iPSCs are more responsive to differentiation to astrocytes than iPSCs derived from wild-type OCRL and that a Lowe syndrome mouse model that lacks OCRL shows a similar pattern. Further, OCRL knockout mice, mutant iPSC-derived iN cells derived from OCRL knockout mice, and the IOB Lowe syndrome mouse model showed a decrease in the activity of the cilia-related sonic hedgehog pathway that organizes the pattern of cellular neuronal differentiation. Our findings suggest mitochondrial dysfunction and impaired ciliary sonic hedgehog signaling as novel mechanisms contributing to altered neuronal differentiation in Lowe syndrome.

Astrocytes play an active role in regulating synapse formation, maturation, and elimination during neuronal development, contributing to the establishment of proper neuronal circuits and brain function (Akdemir et al., 2020; Clavreul et al., 2022; Farhy-Tselnicker and Allen, 2018). Mitochondrial dysfunction in astrocytes leads to abnormal structure and signaling of primary cilia. This dysfunction, which includes depletion of mitochondrial DNA in astrocytes, induces the transcription factors FOXJ1 and RFX, which are master regulators of ciliogenesis, and chronic activation of the mitochondrial integrated stress response (ISRmt) in astrocytes drives anabolic metabolism and is proposed to promote this ciliary elongation (Bear and Caspary, 2023; Ignatenko et al., 2023). It remains unclear, however, whether metabolic ciliopathy is a novel pathogenic mechanism in mitochondria-related neurodegenerative diseases, involving disrupted cilia structure and signaling due to mitochondrial dysfunction in astrocytes.

Shh signaling is crucial for astrocyte development, contributing to the generation of cortical astrocytes. Additionally, it regulates diverse astrocyte functions, including synapse modulation, neuronal activity, and metabolic processes, in a region-specific manner (Garcia, 2021; Gingrich et al., 2022; Hill et al., 2021, 2019). Nevertheless, more research is needed in order to determine whether Shh signaling regulates mitochondrial biogenesis, dynamics, and function in neurons, as well as mediating astrocyte-neuron interactions.

Our findings provide evidence that mitochondrial dysfunction and impairments of the ciliary sonic hedgehog signaling pathway may represent a novel pathogenic mechanism contributing to the abnormal neuronal differentiation observed in Lowe syndrome.

## Materials and methods

### iPSCs culture and reagent

iPSCs were cultured on matrigel (Corning, 354277) in mTeSR1 Plus medium (stem cell technologies, 85850). Media was changed daily, and confluent cells were passaged (1:2) using ReLeSR (stem cell technologies, 05872). All cells were maintained at 37° C, 5% CO_2_.

### Animals

All animal experiments adhered to the guidelines of the Association for Research in Vision and Ophthalmology Statement for the Use of Animals in Ophthalmic and Vision Research and were approved by the Institutional Animal Care and Use Committee (IACUC) of Stanford University School of Medicine. Ocrl^−/−^ Inpp5b^−/−^ INPP5B^+/+^ (IOB) mice were generously provided by Robert L. Nussbaum (University of California, San Francisco). Wild-type (C57BL/6) mice from the Jackson Laboratories were used as controls for the IOB mice. The animals were housed under a 12-hour light/dark cycle with free access to water and food. Mice were anesthetized with isoflurane, with oxygen flow set to 2 liters per minute and isoflurane at 1% delivered via a nose cone.

### Plasmids

The lentiviral vectors for Ngn2-mediated conversion of iPSCs to iN cells is from Thomas C. Sudhof’s lab [27].

### Lentivirus production and infection

5×10^5^ 293FT cells were plated on 60-mm dishes using TurboFect^™^ Transfection Reagent with the following plasmids: 1.5 μg of V-SVG, 2.5 μg pCMV-gag-pol and 3.5 μg of the of lentiviral vector DNA constructs. The supernatant containing viral particles was harvested 48 h after transfection. Virus containing media was passed through a 0.45μm filter (Fisher Scientific, 13–100-105).

### Generation of iN Cells from Human iPSCs

iPSCs were treated with Accutase (stem cell technologies, 07920) and plated as dissociated cells in 24-well plates (iPSCs: 1.5 × 10^4^ cells/well) on day 2 (Figure 1c). Cells were plated on matrigel (Corning, 354277)-coated coverslips in mTeSR1 medium. On day 1, lentivirus prepared as described above (0.3 ml/well of 24-well plate) was added in fresh mTeSR1 medium containing polybrene (8 mg/mL, Sigma). On day 0, the culture medium was replaced with DMEM/F12 (Thermo Fisher Scientific, 11–330-057) containing N2(STEMCELL Technologies, 07152), NEAA (Thermo Fisher Scientific, 11–140-050), human BDNF (10 mg/L, STEMCELL Technologies, 78058), human NT-3 (10 mg/L, PeproTech, 450–03), and mouse laminin (0.2 mg/L, Thermo Fisher Scientific, 23017015). Doxycycline (2 g/L, Fisher Scientific, AC446060050) was added on day 0 to induce TetO gene expression and retained in the medium until the end of the experiment. On day 1, a 24 hr puromycin selection (1 mg/L) period was started. On day 2, replace into Neurobasal medium (Thermo Fisher Scientific, 21103049) supplemented with B27/Glutamax (Invitrogen) containing BDNF and NT3. After day 2, 50% of the medium in each well was exchanged every 2 days. FBS (2.5%) was added to the culture medium on day 10 to support astrocyte viability, and iN cells were assayed on day 14 or 21 in most experiments.

### Immunostaining

Cells were cultured on coverslips coated with 0.1 mg/mL poly-L-lysine and fixed with methanol at −20°C for 15 minutes. The cells were then washed three times with PBS and incubated in a blocking buffer containing 3% bovine serum albumin (w/v) and 0.1% Triton X-100 in PBS for 30 minutes at room temperature (RT). Primary antibodies, diluted in the blocking buffer, were applied for 2 hours at RT. Alexa Fluor 488-, 594-, or 647-conjugated goat secondary antibodies (Thermo Fisher Scientific) were used at a 1:500 dilution and incubated for 1 hour at RT. DNA was stained with 4′,6-diamidino-2-phenylindole (DAPI; Thermo Fisher Scientific). Coverslips were then mounted on slides using ProLong^™^ Gold Antifade mounting medium (Thermo Fisher Scientific). Fluorescent images were captured using an LSM880 Zeiss confocal microscope and processed with ZEN software (Carl Zeiss) or ImageJ software (National Institutes of Health).

### Immunoblotting

Cells were washed twice with ice-cold PBS and lysed in ice-cold RIPA lysis buffer (Millipore, 20–188) containing a protease inhibitors cocktail (Thermo Fisher Scientific, PI78430). The lysate was centrifuged at 13,500 g for 15 minutes at 4°C to remove cell debris. Protein concentrations were measured using the BCA Protein Assay (Thermo Fisher Scientific, 23227). Equal amounts of protein were combined with SDS sample buffer, boiled at 95°C for 5 minutes, and separated by SDS-PAGE. The proteins were then transferred to 0.2 μm nitrocellulose membranes (Bio-Rad, 1620097). The membranes were blocked for 1 hour at room temperature (RT) with 5% non-fat milk in TBS-T (20 mM Tris, pH 7.6, 137 mM NaCl, and 0.1% Tween-20) and incubated overnight at 4°C with primary antibodies in the blocking solution. The membranes were washed three times with TBS-T and incubated with HRP-conjugated anti-mouse or anti-rabbit secondary antibodies (Invitrogen, 31430 and 31460) for 1 hour at RT. After three additional washes with TBS-T, the proteins were visualized using ECL Western blotting substrate (Thermo Fisher Scientific, 34095).

### Primary antibodies

Primary antibodies were obtained from the following sources and used according to the manufacturers’ instructions: mouse IgG1 anti-OCRL/INPP5b, NeuroMab clone N166A/26 (IF 1: 250; UC Davis/NIH NeuroMab Facility), rabbit anti-Nanog (IF 1: 250; 3580S, Cell Signaling Technology), rabbit anti-Oct-4A (C30A3) (IF 1: 250; 2840S, Cell Signaling Technology), Chicken anti-GFAP(IF 1: 250; ab4674, Abcam), mouse IgG2b anti-8-oxo-Dg (IF 1: 250; 4354-MC-050; R&D systems), rabbit anti- Sonic Hedgehog antibody [EP1190Y] (IF: 1:200 WB: 1:500 ab53281, Abcam), rabbit anti- Gli1 antibody (WB: 1:500 ab217326, Abcam), mouse anti-Arl13b antibody (N295B/66) (IF: 1:500 75–287, Antibodies Incorporated), mouse anti- NeuN Antibody, clone A60 (IF: 1:200 MAB377, Sigma-Aldrich), mouse anti-β actin (WB: 1:5000 66009–1, Proteintech)

### RT-PCR

Real-time PCR was conducted using HiScript III RT SuperMix for qPCR plus Grna wiper (Vazyme, R323–01). Real-Time PCR System with the FastSYBR Mixture (2X) (CWBio, CW0955L). The amplification was carried out in 20 μl reaction mixtures containing 100 ng of total DNA, 1X SYBR-Green PCR Master Mix, and 0.5 μM of each primer. Each marker was tested in triplicate reactions in a 96-well plate using a three-step amplification protocol: initial denaturation at 95°C for 5 minutes, followed by 40 cycles of 95°C for 15 seconds, 60°C for 30 seconds, and 72°C for 30 seconds. Data were analyzed using the comparative cycle threshold (Ct) method to determine the relative amounts of rhodopsin and transducin. Relative content was calculated by comparing the Ct values of the rhodopsin and transducin markers to that of the calibrator nuclear marker β-actin. Each measurement was repeated in triplicate, and a non-template control was included in each experiment. The sequences of each gene were supplement figure 1 and 2.

### Oxygen consumption rate (OCR)

Oxygen consumption rate (OCR) was measured using a Seahorse Biosciences XFe96 extracellular flux analyzer. Cells were seeded at a density of 1.25 × 10^5^ cells per well in XFe96 cell culture plates. After 24 hours, cell attachment was confirmed, and the cells were incubated overnight at 37°C with 5% CO2. Prior to the assay, the cells were switched to Seahorse XF DMEM medium containing 1 mM pyruvate, 2 mM glutamine, and 10 mM glucose and equilibrated for 1 hour at 37°C without CO2. OCR was then measured using the following inhibitors: 2.5 μM oligomycin, 2 μM carbonyl cyanide 4-(trifluoromethoxy) phenylhydrazone (FCCP), and 0.5 μM rotenone combined with 0.5 μM antimycin A (Agilent Technologies, 103015–100). Each condition was tested in triplicate cycles, consisting of 3 minutes of mixing followed by 3 minutes of measurement. After the assay, the cell number per well was determined using the Cytation 5, and the OCR was normalized to the cell number for each well.

### Statistical data analysis

All data are presented as mean with standard deviation (SD) from at least 3 independent experiments. Experimental samples and numbers for statistical testing are reported in the corresponding figure legends. All p-values are from Student’s t-tests for two-group comparisons (GraphPad Prism 8).

## Supplementary Material

Supplement 1
