# Supplementary material for: Defective Neurogenesis in Lowe Syndrome is Caused by Mitochondria Loss and Cilia-related Sonic Hedgehog Defects
Source: bioRxiv. 2024 Nov 1:2024.11.01.621496. Preprint. [Version 1] doi: 10.1101/2024.11.01.621496 (PMC11565974; doi:10.1101/2024.11.01.621496)

bioRxiv preprint doi: https://doi.org/10.1101/2024.11.01.621496; this version posted November 2, 2024. The copyright holder for this preprint (which was not certified by peer review) is the author/funder, who has granted bioRxiv a license to display the preprint in perpetuity. It is made available under aCC-BY 4.0 International license.

Supplementary files

Supplementary Figure 1. qPCR primers for iPSCs.

iPSCs-QPCR primer	primer (5' to 3')
Foxg1-F	CCTGCCCTGTGAGTCTTTAAG
Foxg1-R	GTTCACTTACAGTCTGGTCCC
Neun-F	GTAGAGGGACGGAAAATTGAGG
Neun-R	CATAGAATTCAGGCCCGTAGAC
Brn2-F	AAAGTAACTGTCAAATGCGCG
Brn2-R	GCTGTAGTGGTTAGACGCTG
Gfap-F	CCTCCAGCGATTCAACCTTT
Gfap-R	GAAGCTCCAAGATGAAACCAAC
Co2-F	CCCCACATTAGGCTTAAAAACAGAT
Co2-R	TATACCCCCGGTCGTGTAGCGGT
Dloop-F	TATCTTTTGGCGGTATGCACTTTTAACAGT

bioRxiv preprint doi: https://doi.org/10.1101/2024.11.01.621496; this version posted November 2, 2024. The copyright holder for this preprint (which was not certified by peer review) is the author/funder, who has granted bioRxiv a license to display the preprint in perpetuity. It is made available under aCC-BY 4.0 International license.

Dloop-R	TGATGAGATTAGTAGTATGGGAGTGG
Shh-F	CGGAGCGAGGAAGGGAAAG
Shh-R	TTGGGGATAAACTGCTTGTAGGC
Gli1-F	TCTGCCCCCATTGCCCACTTG
Gli1-R	TACATAGCCCCCAGCCCATACCTC
Patch1-F	CGGCGTTCTCAATGGGCTGGTTTT
Patch1-R	GTGGGGCTGCTGTTTCGGGTTCG
Actin-F	TCACCCACACTGTGCCCATCTACGA
Actin-R	CAGCGGAACCGCTCATTGCCAATGG

bioRxiv preprint doi: https://doi.org/10.1101/2024.11.01.621496; this version posted November 2, 2024. The copyright holder for this preprint (which was not certified by peer review) is the author/funder, who has granted bioRxiv a license to display the preprint in perpetuity. It is made available under aCC-BY 4.0 International license.

Supplementary Figure 2. qPCR primers for wild-type and IOB mouse.

WT and IOB mouse -QPCR primer	primer (5' to 3')
neun-F	GTTGCCTACCGGGGTGCACAC
neun-R	TGCTCCAGTGCCGCTCCATAAG
pax6-F	GTTCCCTGTCCTGTGGACTC
pax6-R	ACCGCCCTTGGTTAAAGTCT
brn2-F	AGAGCCCAAGGCAGAAAAGT
brn2-R	GGCGCTCTGGTTAAAGGAG
gfap-F	GGACTGAACCATGTCCTTTGTC
gfap-R	AGGCTAGCTCTATCGGTATAACCTAA
Mito1-F	CTAGAAACCCCGAAACCAAA
Mito1-R	CCAGCTATCACCAAGCTCGT
Cox1-F	TGCTAGCCGCAGGCATTACT
Cox1-R	CGGGATCAAAGAAAGTTGTGT
gli1-F	TTATGGAGCAGCCAGAGAGA
gli1-R	GAGCCCGCTTCTTTGTTAAT
gli2-F	TGAAGGATTCCTGCTCGTG
gli2-R	GAAGTTTTCCAGGACAGAACCA
gli3-F	AAGCGGTCCAAGATCAAGC
gli3-R	TGTTCCTTCCGGCTGTTC
patch1-F	TGACAAAGCCGACTACATGC
patch1-R	AGCGTACTCGATGGGCTCT
gapdh-F	CATCACTGCCACCCAGAAGACTG
gapdh- R	ATGCCAGTGAGCTTCCCGTTCAG

bioRxiv preprint doi: https://doi.org/10.1101/2024.11.01.621496; this version posted November 2, 2024. The copyright holder for this preprint (which was not certified by peer review) is the author/funder, who has granted bioRxiv a license to display the preprint in perpetuity. It is made available under aCC-BY 4.0 International license.

Supplementary Figure 3. Genotyping processing for wild-type and IOB mouse.

(a) Genotyping primers for wild-type and IOB mouse. (b) Genotyping processing for wild-type and IOB mouse.

(a) Genotyping primers

OCRL Wild-type (250 bp)	primer (5' to 3')
GT-F	CCCTTTTCATCTGTTAGGAGAAAT
GT-R	GCATGGTTAAACGCACTATGTGG
**OCRL KO (480 bp)**	**primer (5' to 3')**
OCRL KO –F	GCCCTTTGATTCTAATCCCTTTTCATC
OCRL KO –R	TCTGAGCCCAGAAAGCGAAG

Inpp5b (wt 150 bp/ mut 220 bp)	primer (5' to 3')
E25-F	TAAAGTCTGAAAATCCAAGGC
E34-R	CTCATTTCTCCTTGATTCCAA

INPP5B HumanBAC (240 bp)	primer (5' to 3')
in Exon 1F	CCACCCCACGATTGACTC
in Exon 1R	GGTGTCCCAGCCCTCAG

(b) Genotyping processing

Step 1 - OCRL WT

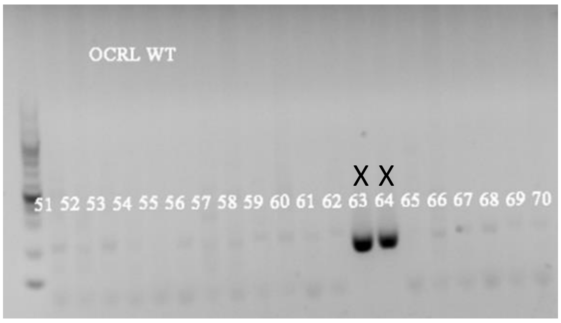

Step 2- OCRL KO

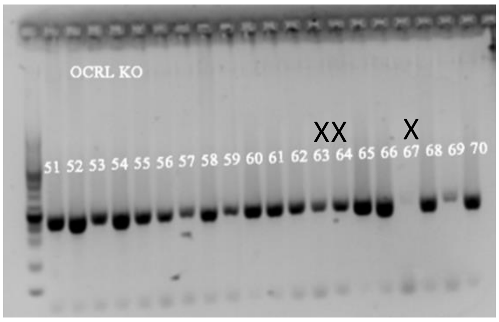

Step 3- Inpp5b (wt 150 bp/ mut 220 bp)

Step 4- INPP5B HumanBAC (240 bp)

bioRxiv preprint doi: https://doi.org/10.1101/2024.11.01.621496; this version posted November 2, 2024. The copyright holder for this preprint (which was not certified by peer review) is the author/funder, who has granted bioRxiv a license to display the preprint in perpetuity. It is made available under aCC-BY 4.0 International license.

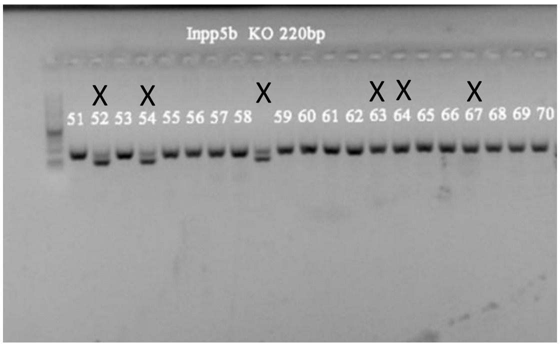

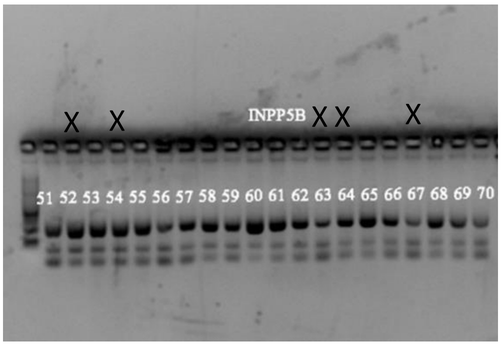

Supplement: Supplement 1 [file NIHPP2024.11.01.621496v1-supplement-1.pdf]
